# Perioperative indocyanine green clearance is predictive for prolonged intensive care unit stay after coronary artery bypass grafting - an observational study

**DOI:** 10.1186/cc8045

**Published:** 2009-09-14

**Authors:** Michael Sander, Claudia D Spies, Katharina Berger, Torsten Schröder, Herko Grubitzsch, Klaus D Wernecke, Christian von Heymann

**Affiliations:** 1 Department of Anaesthesiology and Intensive Care Medicine, Charité Universitätsmedizin - Berlin, Campus Virchow Klinikum and Campus Charité Mitte, Charitéplatz 1, 10117 Berlin, Germany; 2 Department of Cardiovascular Surgery, Charité Universitätsmedizin - Berlin, Campus Charité Mitte, Charitéplatz 1, 10117 Berlin, Germany; 3 SOSTANA GmbH, Wildensteiner Str. 27, 10318 Berlin, Germany

## Abstract

**Introduction:**

During cardiac surgery with cardiopulmonary bypass (CPB) haemodilution occurs. Hepatic dysfunction after CPB is a rare, but serious, complication. Clinical data have validated the plasma-disappearance rate of indocyanine green (PDR ICG) as a marker of hepatic function and perfusion. Primary objective of this analysis was to investigate the impact of haemodilutional anaemia on hepatic function and perfusion by the time course of PDR ICG and liver enzymes in elective CABG surgery. Secondary objective was to define predictors of prolonged ICU treatment like decreased PDR ICG after surgery.

**Methods:**

60 Patients were subjected to normothermic CPB with predefined levels of haemodilution anaemia (haemotacrit (Hct) of 25% versus 20% during CPB). Hepatic function and perfusion was assessed by PDR ICG, plasma levels of aspartate aminotransferase (ASAT) and α-GST. Prolonged ICU treatment was defined as treatment ≥ 48 hours.

**Results:**

Logistic regression analysis showed that all postoperative measurements of PDR ICG (*P *< 0.01), and the late postoperative ASAT (*P *< 0.01) measurement were independent risk factors for prolonged ICU treatment. The predictive capacity for prolonged ICU treatment was best of the PDR ICG one hour after admission to the ICU. Furthermore, the time course of PDR ICG as well as ASAT and α-GST did not differ between groups of haemodilutional anaemia.

**Conclusions:**

Our study provides evidence that impaired PDR ICG as a marker of hepatic dysfunction and hypoperfusion may be a valid marker of prolonged ICU treatment. Additionally this study provides evidence that haemodilutional anaemia to a Hct of 20% does not impair hepatic function and perfusion.

**Trial registration:**

[ISRCTN35655335]

## Introduction

Haemodilution always occurs during cardiac surgery with cardiopulmonary bypass (CPB). Haemodilution reduces blood viscosity and vascular resistance, and may increase large vessel blood flow maintaining whole body oxygen delivery [1]. It appears that the microcirculation can regulate red cell flow and concentration over a wide range of haematocrit (Hct) levels. Hepatic hypoperfusion and ischaemia are rare but severe complications after coronary artery bypass grafting (CABG) [2]. The incidence of hepatic hypoperfusion leading to surgical interventions ranged between 0.2% and 2% in previous investigations [3]. In these patients mortality rises as high as 60% [3]. Inadequate perfusion and oxygenation of the hepatosplachnic system seems to damage the mucosa of the intestine before any other tissue is compromised [4]. There is growing evidence that even transient hepatic hypoperfusion can lead to severe postoperative complications and affect outcome [5]. Immunological cascades resulting in immune paralysis, sepsis and death are thought to be responsible for this negative impact [5-9]. Hepatic hypoperfusion resulting in a decrease in hepatic oxygen delivery seems to be one of the key factors of developing multi-organ dysfunction syndrome [10,11]. Therefore, interest is increasing in monitoring hepatic perfusion in the context of haemodilution in cardiac surgical patients. However, detection of hepatic hypoperfusion is challenging, because there are only a few practicable devices available to gather bedside information in a short period of time. This is, however, crucial as hepatic perfusion and oxygenation is compromised before any systemic sign of hypoperfusion is detected [12]. As standard liver function tests are neither sensitive nor specific to identify patients at risk, a major problem is the early recognition of patients with impaired hepatic function [13]. Therefore, hepatic hypoperfusion and hepatic dysfunction remain disguised for too long in a considerable number of patients. Correction of regional oxygenation and perfusion might be of pivotal relevance to reduce endothelial damage and ischaemia-reperfusion episodes and thus might lower the risk of multi-organ dysfunction syndrome after CPB.

The recent introduction of a new non-invasive method to measure indocyanine green (ICG) plasma disappearance rate (PDR) using pulse densitometry offers an opportunity for the early diagnosis of hepatic dysfunction. Clinical data has validated PDR ICG as a marker of hepatic function and perfusion [14]. Previous studies detected a strong association between PDR ICG and outcome in critically ill patients [15]. Recent data from our group showed that in uncomplicated CABG surgery PDR ICG increases after CPB [16]. Persistent low PDR ICG after surgery might be a hint for impaired hepatic perfusion and might influence outcome. Recently, our group undertook a prospective, randomised and controlled study to investigate oxygen delivery and consumption and the clinical outcome of patients who were randomly allocated to one of two Hct groups (20% or 25%) during normothermic CPB [17]. We report here data from this study regarding hepatic function and perfusion.

The primary aim of this analysis was to investigate hepatic function and perfusion by the time course of the PDR ICG and conventional liver enzymes in different groups of haemodilutional anaemia during CPB in elective CABG surgery. The secondary aim was to assess the predictive capacity of these *a priori *chosen parameters for prolonged intensive care unit (ICU) treatment (≥ 48 hours).

## Materials and methods

### Patients

After institutional approval by the local ethics committee and preoperative written informed consent, 60 patients undergoing elective CABG surgery were considered eligible for this randomised, controlled clinical trial [17]. Randomisation was performed by a computer-generated random list. One patient had to be excluded from analysis as the autologous blood showed multiple clots after CPB and could not be retransfused. Fifty-nine patients, 30 patients in the 25%-Hct group and 29 patients in the 20%-Hct group, remained for statistical analysis.

### Investigational program

Diagnosis, surgery and length of ICU stay were documented for each patient. Vital signs, routine laboratory parameter and complications were recorded on a daily basis. Measurements of the plasma disappearance rate of ICG were performed immediately after induction of anaesthesia, on admission to the ICU, six hours after admission to the ICU and on the first postoperative day. For each measurement, 0.5 mg/kg body weight ICG (Pulsion Medical AG, Munich, Germany) was injected into a central vein. The pulse densitrometric dye decay was analyzed with a commercially available monitor (LiMON-Pulsion Medical AG, Munich, Germany). Each measurement was recorded on a laptop. At the same time points, blood for the analysis of the aspartate aminotransferase (ASAT) and the α-glutathione S-transferase (α-GST) was drawn. For determination of the plasma level of the ASAT and the α-GST 2.7 mL blood was withdrawn. Blood for ASAT was analysed on a commercial laboratory analyser (MODULAR^® ^Analytics D2400, P800, Roche, Germany). The level of α-GST was analysed by enzyme immune assay (Biotrin, Herpkit^®^-Á-GST, Dublin, Ireland) according to the manufacturer's instructions. The team treating the patient in the operating theatre and in the ICU was blinded to the ICG PDR measurements and to the assignment to a haemodilution group to avoid bias.

### Anaesthesia management

After oral premedication with midazolam at 0.1 mg/kg body weight general anaesthesia was introduced in all patients. Intravenous induction was performed with etomidate (0.2 mg/kg) and 5 μg/kg fentanyl, 0.1 mg/kg pancuronium, followed by a continuous infusion of 5 to 10 μg/kg/h fentanyl, repetition boluses of 0.1 mg/kg midazolam and 0.03 mg/kg pancuronium before the start of CPB. Anaesthesia was maintained with 0.6 to 1% of end-tidal volume isoflurane. All patients were ventilated with an oxygen-air mixture (fraction of inspired oxygen (FiO_2_) 0.5) to maintain an end-tidal partial pressure of carbon dioxide (pCO_2_) of 35 to 45 mmHg. Before induction of anaesthesia, haemodynamic monitoring was established with a radial artery catheter for invasive blood pressure monitoring, arterial blood gas sampling and haemoglobin determinations. Heart rate, arterial blood pressure (systolic and diastolic) and central venous pressure were continuously monitored and recorded (Solar 8000; Marquette Hellige, Freiburg, Germany). Arterial oxygen saturation was continuously monitored by pulse oximetry. FiO_2 _and end-tidal isoflurane concentration, as well as end-tidal pCO_2_, were measured (Solar 8000). Additional monitoring in all patients included oesophageal temperature and tidal volume measurements. After orotracheal intubation, a four-lumen central venous catheter (Arrow, Reading, PA, USA) and an introducer sheath for a thermodilution pulmonary artery catheter (8.5 Fr; Arrow, Reading, PA, USA) were inserted routinely into the right internal jugular vein.

### Haemodilution and cardiopulmonary bypass management

Before institution of CPB isovolaemic haemodilution using a hydroxyethylstarch solution 130/0,4 (Voluven^®^, Fresenius-Kabi, Bad Homburg, Germany) was performed to reduce the Hct to a level of 5 ± 1% above the target Hct-level of 20 ± 1% or 25 ± 1%, respectively. For all measurement of Hct and blood lactate, a blood gas analyser (ABL-700 series, Radiometer, Copenhagen, Denmark) was used. This isovolaemic haemodilution was targeted to a Hct level of 20% during CPB in one group of patients and 25% during CPB in the other group of patients.

After sternotomy aprotinin was applied at a dose of 1.5 × 10^6 ^IU (total dose of aprotinin was 50.000 KIU/kg bodyweight including the priming of the CPB). Prior to CPB, 400 U/kg heparin (Liquemin^® ^Hoffmann-La-Roche, Grenzach-Wyhlen, Switzerland) and additional boluses of 50 U/kg were given if necessary to maintain an activated clotting time of at least 480 seconds. Routine CPB priming included HES 10%, balanced electrolyte solution and heparin (8000 U). CPB was performed under normothermic conditions (blood temperature > 35.0°C) using a membrane oxygenator and centrifugal pump flows adjusted to the calculated cardiac index of 2.5 l/min/m^2^. Warm intermittent antegrade blood cardioplegia was used.

### Prediction of prolonged ICU treatment

According to routine clinical practice, all patients are generally transferred from the ICU the day after surgery, if they fulfill the discharge criteria according to standard operating procedures of our department. On average, patients are treated in our department for two days in the ICU [18]. Prolonged ICU treatment was therefore defined as treatment for 48 hours or more. *A priori *we chose age, body mass index (BMI), surgery-related data, group assignment for haemodilutional anaemia and liver perfusion/function parameters to be tested for the ability to predict prolonged ICU treatment.

### ICU treatment/discharge criteria

Indication for ICU treatment in this study was given in all cases of organ dysfunction that were potentially life-threatening, either alone or in combination. This was assumed in the following cases: neurological impairment of different origins (delirium, intoxication, metabolic coma, cerebral insults, elevated intracerebral pressure); respiratory failure with and without hypoxia; cardiogenic failure (including life-threatening arrhythmias); state of shock; severe sepsis; massive bleeding; acute renal failure; or other life-threatening organ dysfunctions.

Patients without any of the above mentioned indications for ICU treatment were transferred within 24 hours postoperatively to the intermediate care unit.

### Statistical analysis

Due to deviations from the normal distribution, all analyses were performed non-parametrically. Results were expressed as median, 25^th ^to 75^th ^percentiles and interquartile ranges. Mann-Whitney-U-test and Fisher's test were used for inter-group differences. Dichotomous variables were examined with the chi-squared test. In case of small samples, larger but unbalanced groups, data sets containing ties, or sparse data, tests were carried out in an exact version. We used a univariate logistic regression model (also exact) to identify parameters that were associated with a prolonged ICU treatment (response: < 48 hours versus ≥ 48 hours). Data with regard to hepatic function and perfusion with respect to time were analysed using nonparametric multivariate analysis of variance for longitudinal data and small sample sizes in a two-factorial design (1^st ^factor (prolonged ICU treatment: < 48 hours versus ≥ 48 hours), 2^nd ^factor (time)) [19]. Therefore, we compared all measurements simultaneously on the corresponding response curves. A *P *< 0.05 was considered to be significant. All tests should be understood as constituting exploratory data analysis, such that no adjustments for multiple testing have been made. Numerical calculations were carried out with SPSS for WINDOWS, Release 14.1 (SPSS Inc, Chicago, IL, USA), LogXact-7 (Cytel Software Corp, Cambridge, MA, USA) and SAS 8.02 (SAS Institute Inc., Cary, NC, USA).

## Results

### Prediction of prolonged ICU treatment

**Figure 1:**
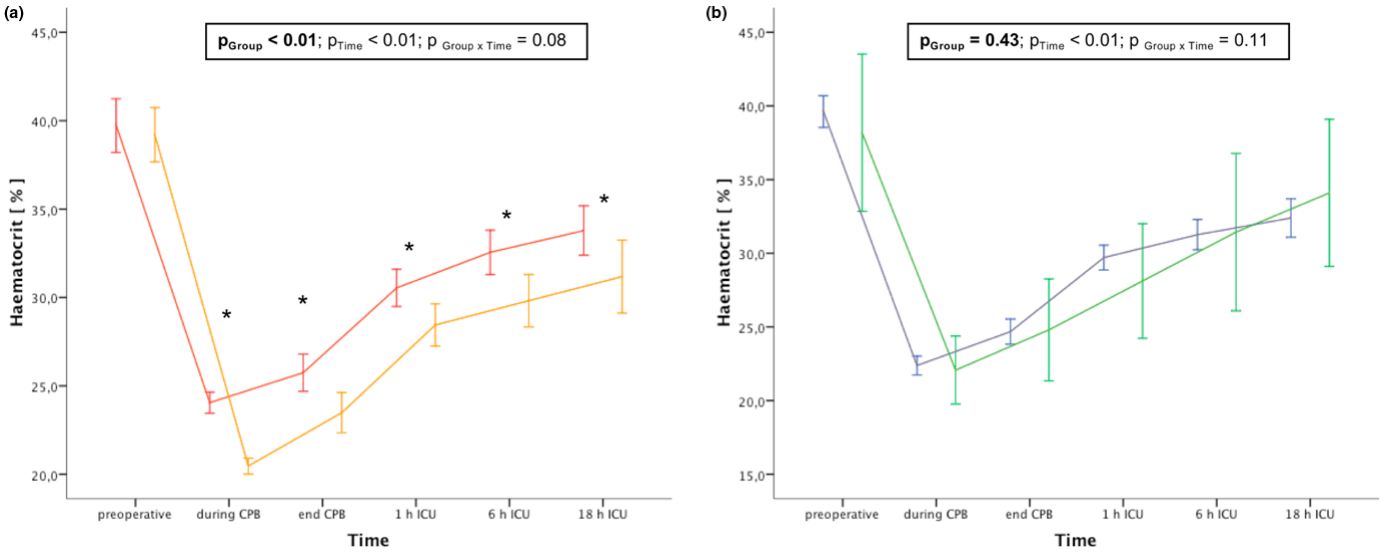
Haemoglobin levels of different patient populations. **(a) **Haemoglobin levels of different groups of haemodilutional anaemia during the study period. red line: 25% hct group, orange line: 20% hct group. **(b) **Haemoglobin levels of patients with and without prolonged intensive care unit (ICU) treatment. blue line: patients with 48 hours or more ICU treatment, green line: patients with less than 48 hours ICU treatment. CPB = cardiopulmonary bypass.

Fifty-three patients were transferred from the ICU within 48 hours. The median ICU treatment in these patients was 22 (20 to 24) hours. Six patients underwent a prolonged ICU stay with a median duration of ICU treatment of 100 (64 to 107) hours. Causes of the prolonged ICU treatment were severe systemic inflammatory response syndrome with vasopressor treatment (n = 3, one patient died later from septic multiple-organ failure), prolonged mechanical ventilation due to severely compromised gas exchange (n = 1), cardiac failure (n = 3) and postoperative symptomatic transitory psychotic syndrome (n = 1). Haemodilutional anaemia did not differ between patients with and without prolonged ICU treatment (Figure 1b).

Univariate analysis of *a priori *chosen parameters for prolonged ICU treatment showed that the group assignment for haemodilutional anaemia (*P *= 0.29), age (*P *= 0.48), BMI (*P *= 0.47), cardiac index after surgery (p_1 h ITS _= 0.86; p_6 h ITS _= 0.46 and p_18 h ITS _= 0.31) and venous oxygen saturation after surgery (p_1 h ITS_= 0.43; p_6 h ITS _= 0.13 and p_18 h ITS _= 0.21) did not influence the length of ICU treatment. Only the postoperative measurements of PDR ICG (p_1 h ITS _< 0.01; p_6 h ITS _= 0.04 and p_18 h ITS _< 0.01) and ASAT (p_1 h ITS _= 0.36; p_6 h ITS _= 0.02 and p_18 h ITS _= 0.01) were identified to influence the length of ICU treatment. Results of the logistic regression analysis of these selected parameters are provided in Table 1.

**Table 1:** Logistic regression of postoperative PDR ICG, ASAT and α-GST

	Coeff. Beta	Lower 95% CI of Beta	Upper 95% CI of Beta	*P *value
**1 hour after admission to ICU**				

PDR ICG (%/min)	***-0.55***	***-1.04***	***-0.21***	*** < 0.01***

ASAT (U/μl)	0.02	-0.03	0.06	0.36

α-GST [U/μl ]	0.02	-0.01	0.04	0.18

**6 hours after admission to ICU**				

PDR ICG (%/min)	***-0.25***	***-0.55***	***-0.01***	***0.04***

ASAT (U/μl)	***0.05***	***0.01***	***0.09***	***0.02***

α-GST (U/μl)	0.07	-0.01	0.15	0.07

**18 hours after admission to ICU**				

PDR ICG (%/min)	***-0.31***	***-0.58***	***-0.08***	*** < 0.01***

ASAT (U/μl)	***0.02***	***0.01***	***0.03***	***0.01***

α-GST (U/μl)	0.33	-0.39	1.03	0.35

α-GST = α-glutathione S-transferase; ASAT = aspartate aminotransferase; CI = confidence interval; ICG = indocyanine green; ICU = intensive care unit; PDR = plasma-disappearance rate.

**Figure 2:**
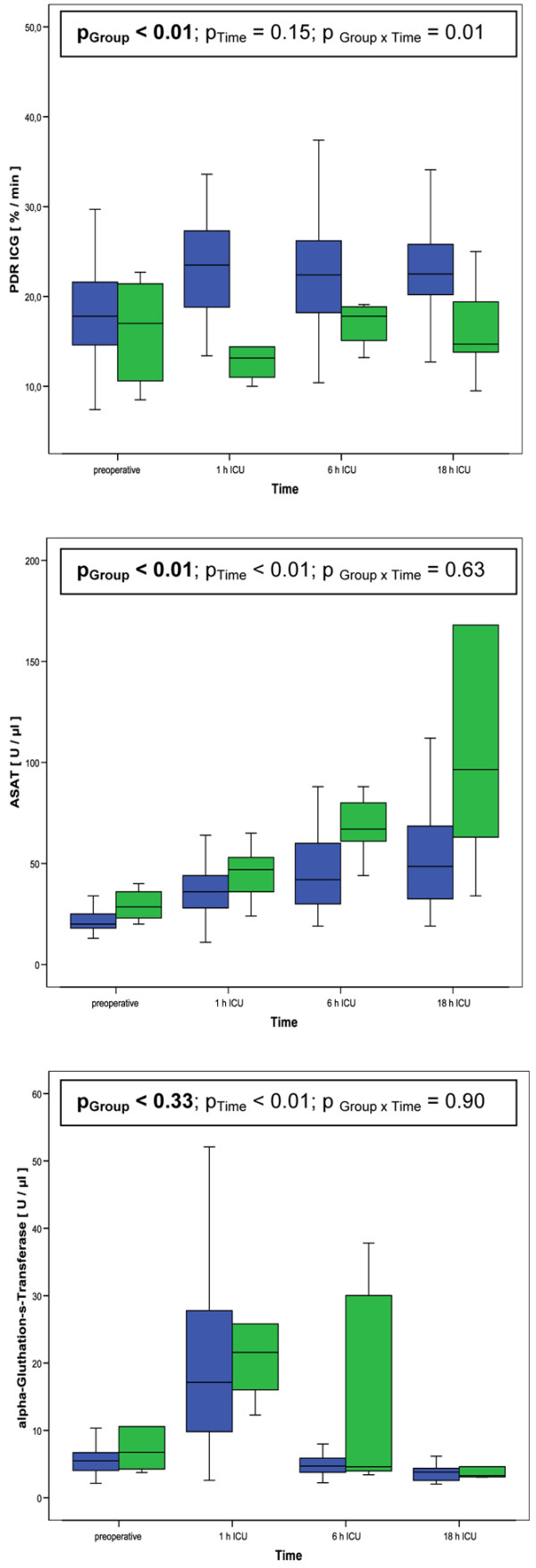
PDR ICG, ASAT and α-GST of patients with (green bars) and without (blue bars) prolonged ICU treatment during the study period. α-GST = α-glutathione-S-transferase; ASAT = aspartate aminotransferase; PDR ICG = plasma disappearance rate of indocyanine green.

Multivariate longitudinal analysis showed that PDR of ICG and ASAT during the whole study period differed significantly between patients being transferred within 48 hours from ICU and patients that were treated longer than 48 hours on the ICU (Figure 2, *P *< 0.01 for PDR ICG and *P *< 0.01 for ASAT).

### Influence of haemodilutional anaemia

**Figure 3:**
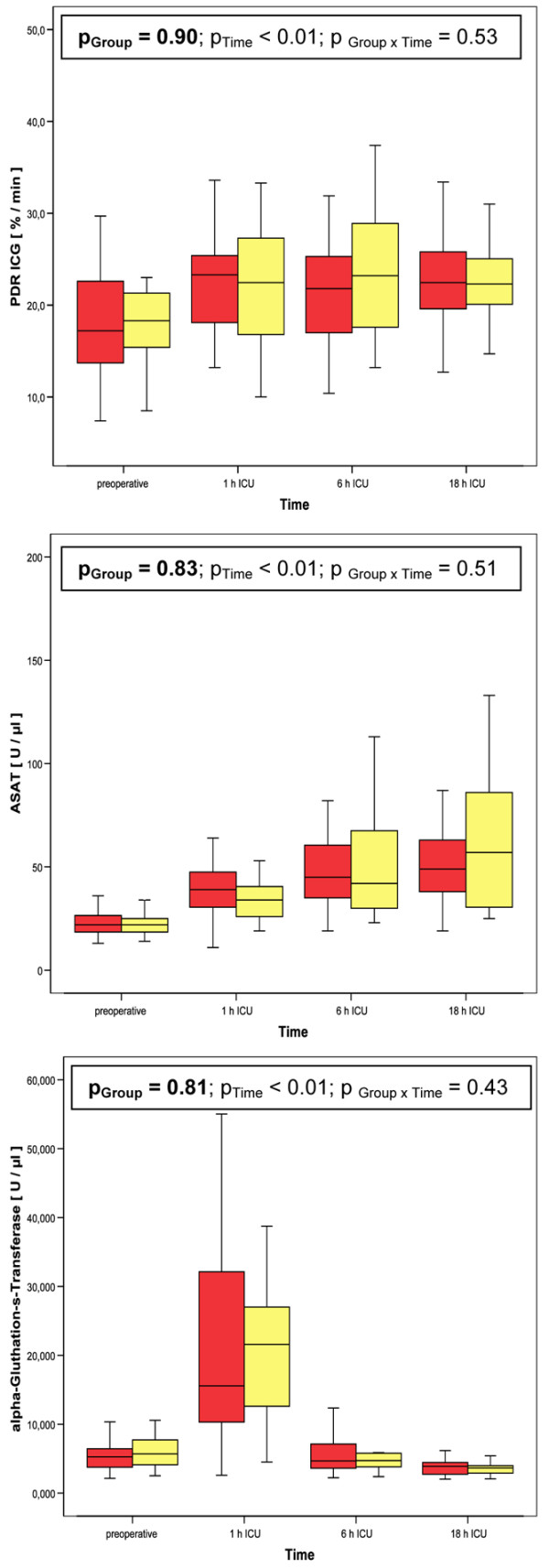
PDR ICG, ASAT and α-GST of the two groups of haemodilutional anaemia during the study period. red bars: haematocrit (Hct) of 25% during cardiopulmonary bypass (CPB). yellow bars: Hct of 20% during CPB. α-GST = α-glutathione-S-transferase; ASAT = aspartate aminotransferase; PDR ICG = plasma disappearance rate of indocyanine green.

Baseline parameters did not differ between study groups of haemodilutional anaemia (Table 2). The targeted level of haemodilutional anaemia was achieved in both study groups (Figure 1a). PDR of ICG as well as ASAT and α-GST did not differ between groups of haemodilutional anaemia (Figure 3). Surgery and ICU-related data are provided in Table 3. There were no significant differences between both groups of haemodilutional anaemia. There were also no significant differences regarding haemodynamics between both groups of haemodilutional anaemia, except systemic vascular resistance one hour after admission to the ICU (Table 4). Two patients in the 25% hct group versus no patient in the 20% hct group received an intra-aortal balloon pump for weaning from CPB. This, however, did not reach statistical significance.

**Table 2:** Baseline characteristics

	haematocrit 25%		haematocrit 20%		*P *value
	
	Median	IQR	Median	IQR	
**Age** **(years)**	60	54-69	64	58-68	0.40

**Gender** **(m - male/f - female)**	26/4		29/0		

**Height** **(m)**	1.76	1.72-1.80	1.75	1.72-1.79	0.81

**Weight** **(kg)**	90	80-100	90	80-101	0.95

**body mass index** **(kg/m^2^)**	28.1	26.1-32.6	28.8	26.9-30.3	0.98

**preoperative haematocrit** **(%)**	41.7	40.1-43	42.2	39.3-45.8	0.72

IQR = interquartile range.

**Table 3:** Intraoperative outcome measures

	haematocrit 25%		haematocrit 20%		*P *value
	
	Median	IQR	Median	IQR	
**CI during CPB** **(l/m**^2^**/min)**	3.2	3.0-3.6	3.2	3.0-3.5	0.40

**Temperature during CPB** **(°C)**	35.5	35.0-35.9	35.7	35.4-36.0	0.21

**Cumulative norepinephrine-dosage during CPB (mg)**	0.06	0.04-0.09	0.05	0.02-0.09	0.41

**Urine volume during CPB** **(mL)**	148	80-250	150	86-177	0.94

**duration of anaesthesia** **(min)**	300	290-320	313	295-338	0.16

**duration of surgery** **(min)**	200	160-220	203	175-243	0.21

**CPB time** **(min)**	74	52-83	71	63-81	0.72

**aortic cross clamp time** **(min)**	45	32-58	44	38-49	0.81

**Length of ICU treatment** **(hours)**	22	20-24	23	21-27	0.29

**Length of mechanical ventilation** **(hours)**	10	8-13	11	10-14	0.10

**APACHE II score**	14	10-19	16	14-17	0.13

APACHE = Acute Physiology and Chronic Health Evaluation; CI = cardiac index; CPB = cardiopulmonary bypass; Hct = haemotacrit; ICU = intensive care unit.

**Table 4:** Haemodynamic measurements throughout the study period

	haematocrit 25%		haematocrit 20%		*P *value
	
	Median	IQR	Median	IQR	
**Before haemodilution**					

MAP (mm Hg)	70	65-79	67	62-78	0.42

CVP (mm Hg)	12	10-15	12	9-14	0.31

CI (l/min/m^2^)	2.2	1.9-2.5	2.1	1.9-2.4	0.39

SVR (dyn/s/cm^-5^)	1045	863-1264	1099	919-1286	0.47

**At the end of surgery**					

MAP (mm Hg)	69	63-87	72	60-80	0.53

CVP (mm Hg)	9	6-12	11	9-14	0.13

CI (l/min/m^2^)	3.6	3.2-4.2	3.7	3.2-4.1	0.46

SVR (dyn/s/cm^-5^)	699	536-811	570	497-788	0.41

**1 hour after admission to ICU**					

MAP (mm Hg)	83	74-92	83	71-89	0.58

CVP (mm Hg)	10	8-13	12	7-14	0.46

CI (l/min/m^2^)	3.0	2.6-3.4	3.2	2.8-3.7	0.24

SVR (dyn/s/cm^-5^)	952	792-1160	798	694-952	0.02

**6 hours after admission to ICU**					

MAP (mm Hg)	79	71-85	73	68-82	0.05

CVP (mm Hg)	10	7-12	11	8-13	0.77

CI (l/min/m^2^)	3.1	2.6-3.6	3.0	2.6-3.6	0.74

SVR (dyn/s/cm^-5^)	870	719-1197	811	611-935	0.26

**18 hours after admission to ICU**					

MAP (mm Hg)	81	73-89	75	69-89	0.54

CVP (mm Hg)	9	5-13	9	8-11	0.63

CI (l/min/m^2^)	3.2	2.8-4.2	3.5	2.9-4.0	0.68

SVR (dyn/s/cm^-5^)	848	694-1022	754	629-963	0.61

CI = cardiac index; CVP = central venous pressure; ICU = intensive care unit; IQR = interquartile range; MAP = mean arterial pressure; SVR = systemic vascular resistance

## Discussion

The most important finding in this study is that the PDR of ICG was the only predictive parameter of prolonged ICU treatment. This was shown in a univariate logistic regression model and a multivariate longitudinal analysis, testing for possible confounders. The second major finding of our investigation was that haemodilutional anaemia during normothermic CPB to a Hct of 0.20 did not impair hepatic perfusion and function and had no impact on postoperative duration of ICU treatment.

Severe hepatic hypoperfusion and dysfunction after cardiac surgery is a rare but often fatal complication. However, mild hypoperfusion with increased hepatic oxygen extraction rate and redistribution of the global cardiac output to other organ systems than the hepatic system has been reported by several investigators [12,20]. Therefore, it seems quite reasonable to monitor hepatic perfusion and function during and after cardiac surgery in the context of haemodilution during normothermic CPB, ICG has been proven by several studies to be a valid marker for liver function and perfusion [21,22]. After intravenous injection, it is bound to plasma proteins and then exclusively eliminated by hepatocytes into the bile. Previous work has established that the PDR ICG is a valid marker of hepatic function and perfusion [23-25].

Haemodilution in this study did not significantly influence haemodynamics, outcome and hepatic function and perfusion parameters after normothermic CPB. Therefore, even under normothermic conditions a Hct of 20% during CPB might be considered safe with regard to functional parameters such as PDR ICG and structural integrity of the liver quantified by ASAT and α-GST. The effect of haemodilution on the perfusion of the hepatic region has been investigated previously. It was demonstrated in an animal study that a haemodilution to a Hct of 0.20 was associated with increased hepatic perfusion and increased ICG clearance [26,27]. Later this was also shown after CABG surgery by the authors' group and others [28]. In our study we found increased levels of ASAT and α-GST after surgery without any difference between groups of haemodilutional anaemia. Increased levels of both enzymes after cardiac surgery have been reported previously [13,20].

Even if haemodilution was not found to be associated with hepatic perfusion and function abnormalities after CABG surgery with CPB in this study, some patients have been reported previously by others to developed postoperative hepatic hypoperfusion and dysfunction quantified by decreased postoperative PDR ICG during and after CABG surgery [12,29]. Therefore, even independent from haemodilution during normothermic CPB, hepatic perfusion might be useful to monitor because it was shown previously that decreased PDR ICG might be of prognostic relevance in other settings [15,23].

Sakka and colleagues concluded in his retrospective study in 336 critically ill patients that PDR ICG as a marker of hepatic perfusion and function is a good predictor of survival in critically ill patients: mortality increased with lower ICG-PDR values, and nonsurvivors had significantly lower ICG-PDR values than survivors [15]. Furthermore in a study in 21 critically ill patients it was concluded that the PDR ICG can identify reversible hepatic dysfunction in septic shock, suggesting good prognosis. Either failure to increase the PDR ICG within 120 hours or an extremely low PDR ICG was a poor prognostic sign [30].

In cardiac surgery, increased proinflammatory responses with an activation of immune cells like macrophages and granulocytes might be involved in postoperative hepatic dysfunction [31]. Keeping in mind that hepatic dysfunction and hypoperfusion was associated with worse outcome in an ICU setting, it seems convincing that early postoperative hepatic dysfunction indicated by a decreased ICG PDR might be associated with prolonged and complicated postoperative ICU treatment. This is in line with our finding that the PDR ICG on admission to the ICU was the best predictor of prolonged ICU treatment.

Evaluation of the plasma concentration of the ASAT has become clinical routine to estimate liver integrity. Cardiac surgery has been shown previously to increase ASAT concentration in the plasma [13]. Different authors concluded that the main reason for the increased ASAT liberation from the hepatocytes might be damage by the systemic inflammation process after CPB and transient hepatic hypoperfusion [32]. Nonetheless, as seen in our study, increases are seen late and are not specific for postoperative complications. This was also reported previously [33]. Another major shortcoming is that increased levels of liver enzymes can only indicate damage and are therefore not suitable to serve as an indication of the patients' regional perfusion. A more sensitive parameter should indicate hepatic hypoperfusion that could be treated before damage to the liver occurs.

Clinically, the α-GST is used to assess postoperative liver damage and liver integrity after liver transplantation [34]. Again the main reason for the increased α-GST levels in the plasma is thought to occur due to systemic inflammation after CPB and transient hepatic hypoperfusion damaging the liver [32]. However, the α-GST, as other standard liver function tests are, is neither sensitive nor specific in the identification of patients with impaired hepatic function [13,35]. Therefore, in a number of patients hepatic hypoperfusion and dysfunction might remain disguised for too long a time period. Significant elevations of α-GST or ASAT point to structural damage to the liver. Yet this is noticed very late after the onset of hepatic hypoperfusion. Thus, detection of elevated liver enzymes cannot be used to prevent damage to the hepatic system - it can only be used to limit damage. In contrast to this, the early determination of the PDR ICG might help to identify patients being at risk of hepatic hypoperfusion and dysfunction that could be treated before structural damage occurs. This would be in accordance to our finding that compared with other variables, an early postoperative decreased PDR ICG was predictive for complicated and prolonged postoperative ICU treatment.

A major limitation of our study is that we cannot provide a causal relationship between the decreased PDR ICG and the observed prolonged ICU treatment. However, this should be done by goal-directed study aiming at improving the PDR ICG in patients at risk of hepatic hypoperfusion after cardiac surgery. Another limitation is the rather small sample size of patients with prolonged ICU treatment. Therefore, the impact of the PDR ICG on outcome should be studied in a group of patients with higher perioperative risk for complications. In this study all patients received aprotinin as our standard antifibrinolytic therapy at that time. This was performed because the evidence of the potential deleterious effects of aprotinin was not published at the time at which this study was performed. Nevertheless, as all patients were treated with aprotinin this should not influence our results.

## Conclusions

In conclusion this study found evidence that impaired hepatic function and perfusion quantified by measurements of PDR ICG was an early marker of prolonged ICU treatment. Therefore, PDR ICG might serve as an early marker for compromised regional perfusion and goal-directed strategies aimed at improving the PDR ICG should be undertaken in cardiac surgical patients at risk to determine whether an outcome benefit exists. Furthermore, our study of patients undergoing different levels of haemodilutional anaemia investigating hepatic function and perfusion showed that we could not observe significant differences between hepatic function and perfusion quantified by measurements of PDR ICG, plasma levels of ASAT and α-GST. Therefore, haemodilution during CPB to 20% might be considered safe with regard to hepatic perfusion and function in patients with no pre-existing gastrointestinal diseases.

## Key messages

• Our study showed that impaired PDR ICG as a marker of hepatic function and perfusion may be a valid marker of prolonged ICU treatment.

• Our study provides evidence that haemodilutional anaemia to a Hct of 20% does not impair hepatic function and perfusion.

## Abbreviations

α-GST: α-glutathione S-transferase; ASAT: aspartate aminotransferase; BMI: body mass index; CABG: coronary artery bypass graft; CPB: cardiopulmonary bypass; FiO_2_: fraction of inspired oxygen; Hct: haematocrit; ICG: indocyanine green; ICU: intensive care unit; pCO_2_: partial pressure of carbon dioxide; PDR: plasma-disappearance rate.

## Competing interests

The authors declare that they have no competing interests.

## Authors' contributions

MS and CvH prepared the manuscript, carried out the measurements, conceived the study and performed the statistical analysis. KDW prepared the statistical part of the manuscript and performed the statistical analysis. KB und TS helped with the drafting of the manuscript. HG helped with the recruitment of patients. CS drafted the manuscript, helped with the study design and coordination. All authors read and approved the final manuscript.

## References

[B1] Gordon RJ Ravin M Rawitscher RE Daicoff GR Changes in arterial pressure, viscosity and resistance during cardiopulmonary bypass J Thorac Cardiovasc Surg 1975 69 552 561 1117744

[B2] Bach F Silomon M Grundmann U Sturner J Graeter T Larsen R Splanchnikusperfusion unter Dopexamin bei kardiochirurgischen Eingriffen Anaesthesist 1999 48 713 717 10.1007/s001010050775 10551920

[B3] Ott MJ Buchman TG Baumgartner WA Postoperative abdominal complications in cardiopulmonary bypass patients: a case-controlled study Ann Thorac Surg 1995 59 1210 1213 10.1016/0003-4975(95)00133-6 7733723

[B4] Dantzker DR The gastrointestinal tract. The canary of the body? JAMA 1993 270 1247 1248 10.1001/jama.270.10.1247 8355390

[B5] Meier-Hellmann A Reinhart K Bredle DL Sakka SG Therapeutic options for the treatment of impaired gut function J Am Soc Nephrol 2001 12 Suppl 17 S65 S69 11251035

[B6] Tamion F Richard V Sauger F Menard JF Girault C Richard JC Thuillez C Leroy J Bonmarchand G Gastric mucosal acidosis and cytokine release in patients with septic shock Crit Care Med 2003 31 2137 2143 10.1097/01.CCM.0000079600.49048.28 12973171

[B7] Poeze M Ramsay G Buurman WA Greve JW Dentener M Takala J Increased hepatosplanchnic inflammation precedes the development of organ dysfunction after elective high-risk surgery Shock 2002 17 451 458 10.1097/00024382-200206000-00002 12069179

[B8] Fink MP Adequacy of gut oxygenation in endotoxemia and sepsis Crit Care Med 1993 21 S4 S8 8428495 10.1097/00003246-199302001-00002

[B9] Siems W Kowalewski J Werner A Schimke I Gerber G Radical formation in the rat small intestine during and following ischemia Free Radic Res Commun 1989 7 347 353 10.3109/10715768909087961 2583551

[B10] Kern H Schroder T Kaulfuss M Martin M Kox WJ Spies CD Enoximone in contrast to dobutamine improves hepatosplanchnic function in fluid-optimized septic shock patients Crit Care Med 2001 29 1519 1525 10.1097/00003246-200108000-00004 11505119

[B11] Lehmann C Taymoorian K Wauer H Krausch D Birnbaum J Kox WJ Effects of the stable prostacyclin analogue iloprost on the plasma disappearance rate of indocyanine green in human septic shock Intensive Care Med 2000 26 1557 1560 10.1007/s001340000662 11126272

[B12] Braun JP Schroeder T Buehner S Dohmen P Moshirzadeh M Grosse J Streit F Schlaefke A Armstrong VW Oellerich M Lochs H Konertz W Kox WJ Spies C Splanchnic oxygen transport, hepatic function and gastrointestinal barrier after normothermic cardiopulmonary bypass Acta Anaesthesiol Scand 2004 48 697 703 10.1111/j.1399-6576.2004.00392.x 15196101

[B13] Kumle B Boldt J Suttner SW Piper SN Lehmann A Blome M Influence of prolonged cardiopulmonary bypass times on splanchnic perfusion and markers of splanchnic organ function Ann Thorac Surg 2003 75 1558 1564 10.1016/S0003-4975(02)04903-2 12735579

[B14] Birnbaum J Lehmann C Taymoorian K Krausch D Wauer H Grundling M Spies C Kox WJ [The effect of dopexamine and iloprost on plasma disappearance rate of indocyanine green in patients in septic shock] Anaesthesist 2003 52 1014 1019 10.1007/s00101-003-0580-1 14992087

[B15] Sakka SG Reinhart K Meier-Hellmann A Prognostic value of the indocyanine green plasma disappearance rate in critically ill patients Chest 2002 122 1715 1720 10.1378/chest.122.5.1715 12426276

[B16] Sander M Spies CD Foer A Syn DY Grubitzsch H Von HC Peri-operative plasma disappearance rate of indocyanine green after coronary artery bypass surgery Cardiovasc J Afr 2007 18 375 379 18092113 PMC4170503

[B17] von Heymann C Sander M Foer A Heinemann A Spiess B Braun J Kramer M Grosse J Dohmen P Dushe S Halle J Konertz WF Wernecke KD Spies C The impact of an hematocrit of 20% during normothermic cardiopulmonary bypass for elective low risk coronary artery bypass graft surgery on oxygen delivery and clinical outcome - a randomized controlled study [ISRCTN35655335] Crit Care 2006 10 R58 10.1186/cc4891 16606474 PMC1550910

[B18] Hein OV Birnbaum J Wernecke KD Konertz W Jain U Spies C Three-year survival after four major post-cardiac operative complications Crit Care Med 2006 34 2729 2737 10.1097/01.CCM.0000242519.71319.AD 16971859

[B19] Brunner E Domhof S Langer F Non-parametric analysis of longitudinal data in factorial experiments 2002 New York: John Wiley & Sons

[B20] Jakob SM Ruokonen E Takala J Assessment of the adequacy of systemic and regional perfusion after cardiac surgery Br J Anaesth 2000 84 571 577 10844831 10.1093/bja/84.5.571

[B21] Autschbach R Falk V Lange H Oellerich M Walther T Mohr FW Dalichau H Assessment of metabolic liver function and hepatic blood flow during cardiopulmonary bypass Thorac Cardiovasc Surg 1996 44 76 80 10.1055/s-2007-1011990 8782332

[B22] Uusaro A Ruokonen E Takala J Gastric mucosal pH does not reflect changes in splanchnic blood flow after cardiac surgery Br J Anaesth 1995 74 149 154 10.1093/bja/74.2.149 7696062

[B23] Kimura S Yoshioka T Shibuya M Sakano T Tanaka R Matsuyama S Indocyanine green elimination rate detects hepatocellular dysfunction early in septic shock and correlates with survival Crit Care Med 2001 29 1159 1163 10.1097/00003246-200106000-00014 11395594

[B24] Maynard ND Bihari DJ Dalton RN Beale R Smithies MN Mason RC Liver function and splanchnic ischemia in critically ill patients Chest 1997 111 180 187 10.1378/chest.111.1.180 8996014

[B25] Sakka S Meier-Hellmann A Indocyanin Green for Assessment of liver function in Critically Ill Patients Yearbook of Intensive Care and Emergency Medicine 2001 2001 Berlin-Heidelberg-New York: Springer Verlag

[B26] Habler O Kleen M Hutter J Podtschaske A Tiede M Kemming G Corso C Batra S Keipert P Faithfull S Messmer K Effects of hemodilution on splanchnic perfusion and hepatorenal function. II. Renal perfusion and hepatorenal function Eur J Med Res 1997 2 419 424 9348268

[B27] Kleen M Habler O Hutter J Podtschaske A Tiede M Kemming G Corso C Batra S Keipert P Faithfull S Messmer K Effects of hemodilution on splanchnic perfusion and hepatorenal function. I. Splanchnic perfusion Eur J Med Res 1997 2 413 418 9348267

[B28] Mathie RT Ohri SK Batten JJ Peters AM Keogh BE Hepatic blood flow during cardiopulmonary bypass operations: the effect of temperature and pulsatility J Thorac Cardiovasc Surg 1997 114 292 293 10.1016/S0022-5223(97)70162-4 9270653

[B29] Kramer WG Romagnoli A Effect of surgery and cardiopulmonary bypass on indocyanine green pharmacokinetics Tex Heart Inst J 1986 13 77 82 15226835 PMC324601

[B30] Kimura S Yoshioka T Shibuya M Sakano T Tanaka R Matsuyama S Indocyanine green elimination rate detects hepatocellular dysfunction early in septic shock and correlates with survival Crit Care Med 2001 29 1159 1163 10.1097/00003246-200106000-00014 11395594

[B31] Koo DJ Chaudry IH Wang P Kupffer cells are responsible for producing inflammatory cytokines and hepatocellular dysfunction during early sepsis J Surg Res 1999 83 151 157 10.1006/jsre.1999.5584 10329110

[B32] Boyle EM Jr Pohlman TH Johnson MC Verrier ED Endothelial cell injury in cardiovascular surgery: the systemic inflammatory response Ann Thorac Surg 1997 63 277 284 10.1016/S0003-4975(96)01061-2 8993292

[B33] Olsson R Hermodsson S Roberts D Waldenstrom J Hepatic dysfunction after open-heart surgery Scand J Thorac Cardiovasc Surg 1984 18 217 222 6152078 10.3109/14017438409109894

[B34] Chouker A Martignoni A Schauer RJ Dugas M Schachtner T Kaufmann I Setzer F Rau HG Lohe F Jauch KW Peter K Thiel M Alpha-gluthathione S-transferase as an early marker of hepatic ischemia/reperfusion injury after liver resection World J Surg 2005 29 528 534 10.1007/s00268-004-7431-3 15776301

[B35] McSweeney ME Garwood S Levin J Marino MR Wang SX Kardatzke D Mangano DT Wolman RL Adverse gastrointestinal complications after cardiopulmonary bypass: can outcome be predicted from preoperative risk factors? Anesth Analg 2004 98 1610 7 table. 10.1213/01.ANE.0000113556.40345.2E 15155313

